# *catena*-Poly[[diaquaterbium(III)]-tetrakis(μ_2_-pyridine-4-carboxylato-κ^2^ *O*:*O*′)-[diaquaterbium(III)]-bis(μ_2_-pyridine-4-carboxylato-κ^2^ *O*:*O*′)]

**DOI:** 10.1107/S1600536811038487

**Published:** 2011-09-30

**Authors:** Xiangfei Zhang, Bin Zhai

**Affiliations:** a Department of Chemistry, Shangqiu Normal University, Shangqiu 476000 Henan, People’s Republic of China

## Abstract

The title complex, [Tb_2_(C_6_H_4_NO_2_)_6_(H_2_O)_4_]_*n*_, was isolated under hydro­thermal conditions using the ligand pyridine-4-carb­oxy­lic acid (H*L*) and Tb_2_O_3_. The deprotonated *L* ^2−^ ligands adopt bridging coordination modes. The central Tb^III^ atom is bridged by *L* ^2−^ ligands, forming a polymeric chain parallel to the *a* axis. Supra­molecular O—H⋯N inter­actions link the chains, building up a layer parallel to (010). O—H⋯O hydrogen bonds also occur. Two of the pyridine rings are disordered by rotation around the central C—N direction with occupancy ratios of 0.53 (1):0.47 (1).

## Related literature

For the properties of metal-organic coordination polymers, see: Bradshaw *et al.* (2004 ▶); Singh & Roesky (2007 ▶); Rosi *et al.* (2002 ▶); Thirumurugan & Natarajan (2005 ▶); Thirumurugan *et al.* (2008 ▶); Forster & Cheetham (2002 ▶); Fan & Zhu (2007 ▶).
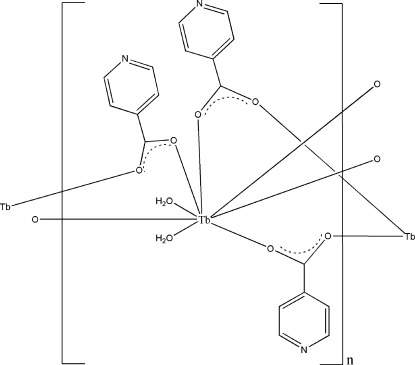


## Experimental

### 

#### Crystal data

[Tb_2_(C_6_H_4_NO_2_)_6_(H_2_O)_4_]
*M* *_r_* = 1122.52
Monoclinic, 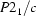

*a* = 9.7008 (10) Å
*b* = 19.813 (2) Å
*c* = 11.6253 (12) Å
β = 112.009 (1)°
*V* = 2071.6 (4) Å^3^
*Z* = 2
Mo *K*α radiation
μ = 3.46 mm^−1^
*T* = 293 K
0.26 × 0.20 × 0.18 mm

#### Data collection

Bruker SMART CCD area-detector diffractometer
Absorption correction: multi-scan (*SADABS*; Sheldrick, 2008*a* ▶) *T*_min_ = 0.466, *T*_max_ = 0.575
11208 measured reflections
4055 independent reflections
2998 reflections with *I* > 2σ(*I*)
*R*_int_ = 0.026

#### Refinement

*R*[*F* ^2^ > 2σ(*F* ^2^)] = 0.035
*wR*(*F* ^2^) = 0.105
*S* = 1.11
4055 reflections
259 parameters
148 restraints
H-atom parameters constrained
Δρ_max_ = 0.78 e Å^−3^
Δρ_min_ = −1.60 e Å^−3^

### 

Data collection: *SMART* (Bruker, 1997 ▶); cell refinement: *SAINT* (Bruker, 1997 ▶); data reduction: *SAINT*; program(s) used to solve structure: *SHELXS97* (Sheldrick, 2008*b* ▶); program(s) used to refine structure: *SHELXL97* (Sheldrick, 2008*b* ▶); molecular graphics: *PLATON* (Spek, 2009 ▶); software used to prepare material for publication: *publCIF* (Westrip, 2010 ▶).

## Supplementary Material

Crystal structure: contains datablock(s) I. DOI: 10.1107/S1600536811038487/dn2711sup1.cif

Structure factors: contains datablock(s) I. DOI: 10.1107/S1600536811038487/dn2711Isup2.hkl

Additional supplementary materials:  crystallographic information; 3D view; checkCIF report

## Figures and Tables

**Table 1:** Hydrogen-bond geometry (Å, °)

*D*—H⋯*A*	*D*—H	H⋯*A*	*D*⋯*A*	*D*—H⋯*A*
O7—H71⋯N3^i^	0.84	1.96	2.787 (7)	168
O7—H72⋯N2^ii^	0.84	1.98	2.802 (5)	167
O8—H81⋯N1^iii^	0.85	2.01	2.837 (7)	163
O8—H82⋯O7^iv^	0.85	2.16	3.002 (5)	171

Symmetry codes: (i) 
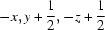
; (ii) 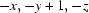; (iii) 
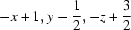
; (iv) 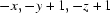.

**Fig. 1.**
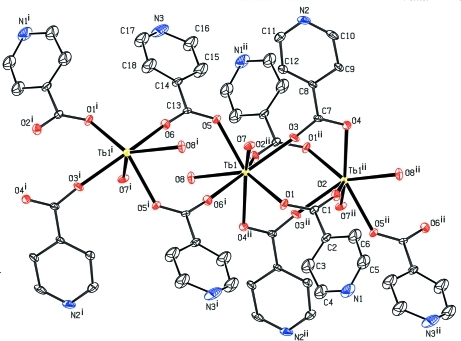
Partial view of the polymeric chain. Ellipsoids are drawn at the 30% probability level. H atoms and one component of the disordered pyridine rings have been omitted for clarity. [Symmetry codes: (i) -x, -y+1, -z+1; (ii) -x+1, -y+1, -z+1]

**Fig. 2.**
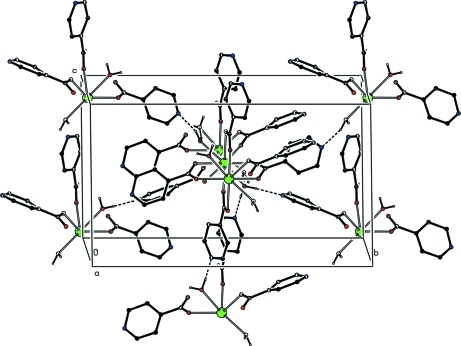
Partial packing view showing the formation of layer through O-H···N hydrogen bonds. H atoms not involved in hydrogen bondings have been omitted for clarity.

## supplementary crystallographic information

### Comment

Nowadays the connection of metal-organic coordination polymers based on transition metals and multifunctional bridging ligands has proven to be a promising field due to their intriguing and beautiful topologies and potential functions in material chemistry (Singh & Roesky, 2007; Forster & Cheetham, 2002 ). Great effort has been devoted to prepare such materials (Rosi *et al.*, 2002; Thirumurugan & Natarajan, 2005; Fan & Zhu, 2007; Bradshaw *et al.*, 2004; Thirumurugan *et al.*, 2008), for example, multidentate aromatic polycarboxylic acids including benzene-1,2-dicarboxylate, benzene-1,3-dicarboxylate, benzene-1,4-dicarboxylate, benzene-1,2,3-tricarboxylate, benzene-1,2,4-tricarboxylate, benzene-1,3,5- tricarboxylate and benzene-1,2,4,5-tetracarboxylate have been widely used for the syntheses of coordination polymers of transition metal ions, and the syntheses are mainly through the direct interaction between the metal ions and carboxylate groups to construct one-, two-, and three-dimensional networks in a variety of coordination modes. In general, carboxylate positions, functional groups and ligand conformations are important for syntheses of these hybrid materials. The ligand pyridine-4-carboxylic acid (H_2_L) has proven to be a good building block for constructing functional coordination polymers. On the other hand, Tb^3+^ complex is strongly fluorescent, having a large fluorescence quantum yield and very long fluorescence lifetime.

In compound I, each Tb^III^ atom is bonded to six Oatoms from six different carboxylates (Tb–O = 2.424 (3)–2.491 (4) Å) and two water molecules (Tb–O_w_ = 2.531 (3)–2.559 (3) Å). The coordination geometry around Tb atom can also be described as a distorted square antiprism with O–Tb–O bond angles ranging from 70.52 (11) to 147.15 (11)°. Adjacent two Tb centers are connected together via four bridging carboxylates with the Tb···Tb distances of 4.349 Å, which are further extended by other two L^2- ^ligand to generate an infinite chain (Figure 1) which develop parallel to the a axis. All carboxylate groups in (I) adopt bridging modes and the potential N-donor coordination site still remains free.

O-H···N supramolecular interactions link the chain to form layers parallel to the (0 1 0) plane (Table 1, Fig. 2). There are also O-H···O intramolecular hydrogen bonds within the chains (Table 1).

### Experimental

Synthesis of [TbL_3_(H_2_O)_2_]_n_ (I). A mixture of H_2_L (0.3 mmol), Tb_2_O_3_(0.1 mmol) and H_2_O (15 mL) were placed in a 25 mL Teflon-lined steel vessel and heated to 180 °C for 5 days, then cooled to room temperature. The resulting colorless block-shaped crystals of (I) were washed several times by water and diethyl ether. Elemental analysis calcd for (I) (%): C, 38.52; H, 2.87; N, 7.49. Found: C, 38.46; H, 2.94; N,7.45. Selected IR spectra for (I): ν (cm^-1^) = 3449 s, 1632 s, 1401 m,745 m.

### Refinement

Two of the pyridine rings were disordered by slight rotation around the axial C—N bond. The occupancy factors of the two positions were refined restraining the sum of the occupancy factors to be equal to 1. The values obtained after refinement are 0.47 (1) and 0.53 (1). These values were then fixed and the anisotropic thermal parameters were introduced with EADP restraints (Sheldrick, 2008).

H atoms bound to C atoms were placed geometrically and treated as riding with C-H = 0.93 Å and U_iso_ = 1.2U_eq_(C). H atoms of water molecule were located in difference Fourier maps and included in the subsequent refinement using restraints (O-H= 0.85 (1)Å and H···H= 1.40 (2)Å) with U_iso_(H) = 1.5U_eq_(O). In the last cycles of refinement they were considered as riding on their parent O atoms.

### Crystal data

**Table:**

[Tb_2_(C_6_H_4_NO_2_)_6_(H_2_O)_4_]	*F*(000) = 1096
*M**_r_* = 1122.52	*D*_x_ = 1.800 Mg m^−^^3^
Monoclinic, *P*2_1_/*c*	Mo *K*α radiation, λ = 0.71073 Å
Hall symbol: -P 2ybc	Cell parameters from 5467 reflections
*a* = 9.7008 (10) Å	θ = 2.2–26.4°
*b* = 19.813 (2) Å	µ = 3.46 mm^−^^1^
*c* = 11.6253 (12) Å	*T* = 293 K
β = 112.009 (1)°	Block, colourless
*V* = 2071.6 (4) Å^3^	0.26 × 0.20 × 0.18 mm
*Z* = 2	

### Data collection

**Table:**

Bruker SMART CCD area-detector diffractometer	4055 independent reflections
Radiation source: fine-focus sealed tube	2998 reflections with *I* > 2σ(*I*)
graphite	*R*_int_ = 0.026
φ and ω scans	θ_max_ = 26.0°, θ_min_ = 2.3°
Absorption correction: multi-scan (*SADABS*; Sheldrick, 2008)	*h* = −11→11
*T*_min_ = 0.466, *T*_max_ = 0.575	*k* = −10→24
11208 measured reflections	*l* = −14→14

### Refinement

**Table:**

Refinement on *F*^2^	Primary atom site location: structure-invariant direct methods
Least-squares matrix: full	Secondary atom site location: difference Fourier map
*R*[*F*^2^ > 2σ(*F*^2^)] = 0.035	Hydrogen site location: inferred from neighbouring sites
*wR*(*F*^2^) = 0.105	H-atom parameters constrained
*S* = 1.11	*w* = 1/[σ^2^(*F*_o_^2^) + (0.0526*P*)^2^ + 5.420*P*] where *P* = (*F*_o_^2^ + 2*F*_c_^2^)/3
4055 reflections	(Δ/σ)_max_ = 0.012
259 parameters	Δρ_max_ = 0.78 e Å^−^^3^
148 restraints	Δρ_min_ = −1.60 e Å^−^^3^

### Special details

**Table:**

Geometry. All esds (except the esd in the dihedral angle between two l.s. planes) are estimated using the full covariance matrix. The cell esds are taken into account individually in the estimation of esds in distances, angles and torsion angles; correlations between esds in cell parameters are only used when they are defined by crystal symmetry. An approximate (isotropic) treatment of cell esds is used for estimating esds involving l.s. planes.
Refinement. Refinement of F^2^ against ALL reflections. The weighted R-factor wR and goodness of fit S are based on F^2^, conventional R-factors R are based on F, with F set to zero for negative F^2^. The threshold expression of F^2^ > 2sigma(F^2^) is used only for calculating R-factors(gt) etc. and is not relevant to the choice of reflections for refinement. R-factors based on F^2^ are statistically about twice as large as those based on F, and R- factors based on ALL data will be even larger. The ISOR instruction are used for C16 C16' C17' C5 C5' C15 C15' C18' atoms to insolve their ADP alerts. The instructions of DFIX and DANG are used for H atoms on O7 and O8 water molecules, in order to place H atoms of water molecules in calculated positions as rigiding atoms. The SIMU and DELU instructions against C16 N3 C13 O6 C1 O2 atoms are used for insolveing their ADP alerts. In addition, the disordered C3 C4 C5 C6 and C3' C4' C5' C6' atoms are localized by the differece Fourier map, which are treated as disordered part with 0.5 occupancy. Whereas, the C15 C16 C17 C18 and C15' C16' C17' C18' atoms are treated as disordered part with free refinement.

### Fractional atomic coordinates and isotropic or equivalent isotropic displacement parameters (Å^2^)

**Table:**

	*x*	*y*	*z*	*U*_iso_*/*U*_eq_	Occ. (<1)
Tb1	0.28030 (2)	0.499202 (11)	0.50935 (2)	0.01827 (11)	
O1	0.3953 (4)	0.61276 (17)	0.5299 (3)	0.0248 (8)	
O2	0.5886 (4)	0.60949 (17)	0.4690 (3)	0.0270 (8)	
O3	0.3869 (4)	0.49073 (17)	0.3515 (3)	0.0253 (8)	
O4	0.5557 (4)	0.49956 (19)	0.2667 (4)	0.0324 (10)	
O5	0.0756 (4)	0.43450 (17)	0.3615 (3)	0.0235 (8)	
O6	−0.1256 (4)	0.43637 (18)	0.4071 (3)	0.0274 (8)	
O7	0.1132 (4)	0.57515 (17)	0.3392 (3)	0.0262 (8)	
H71	0.1377	0.6148	0.3290	0.039*	
H72	0.0602	0.5633	0.2667	0.039*	
O8	0.1792 (4)	0.41684 (18)	0.6284 (3)	0.0267 (8)	
H81	0.2344	0.3999	0.6975	0.040*	
H82	0.0949	0.4233	0.6325	0.040*	
N2	0.1002 (5)	0.4546 (3)	−0.1043 (4)	0.0355 (12)	
C1	0.5085 (5)	0.6369 (2)	0.5182 (4)	0.0191 (10)	
C2	0.5552 (6)	0.7071 (3)	0.5691 (5)	0.0263 (11)	
C3	0.5412 (18)	0.7272 (6)	0.6767 (14)	0.065 (2)	0.53
H3	0.4991	0.6988	0.7183	0.078*	0.53
C4	0.5922 (18)	0.7922 (6)	0.7231 (14)	0.065 (2)	0.53
H4	0.5850	0.8049	0.7976	0.078*	0.53
N1	0.6468 (7)	0.8341 (3)	0.6704 (7)	0.0652 (19)	
C5	0.6418 (18)	0.8184 (6)	0.5577 (13)	0.065 (2)	0.53
H5	0.6608	0.8519	0.5093	0.078*	0.53
C6	0.6088 (18)	0.7530 (6)	0.5098 (14)	0.065 (2)	0.53
H6	0.6236	0.7410	0.4379	0.078*	0.53
C3'	0.4669 (18)	0.7485 (7)	0.6043 (18)	0.065 (2)	0.47
H3'	0.3716	0.7349	0.5949	0.078*	0.47
C4'	0.5197 (17)	0.8116 (7)	0.6545 (18)	0.065 (2)	0.47
H4'	0.4566	0.8389	0.6778	0.078*	0.47
C5'	0.7305 (18)	0.7961 (7)	0.6254 (18)	0.065 (2)	0.47
H5'	0.8200	0.8139	0.6273	0.078*	0.47
C6'	0.6903 (17)	0.7308 (7)	0.5758 (18)	0.065 (2)	0.47
H6'	0.7527	0.7052	0.5490	0.078*	0.47
C7	0.4248 (6)	0.4912 (2)	0.2593 (5)	0.0205 (11)	
C8	0.3085 (5)	0.4796 (3)	0.1332 (4)	0.0201 (10)	
C9	0.3287 (7)	0.5057 (3)	0.0274 (5)	0.0286 (12)	
H9A	0.4113	0.5317	0.0344	0.034*	
C10	0.2195 (7)	0.4908 (3)	−0.0881 (5)	0.0353 (15)	
H10A	0.2319	0.5076	−0.1583	0.042*	
C11	0.0841 (6)	0.4311 (3)	−0.0041 (5)	0.0392 (15)	
H11A	0.0013	0.4045	−0.0146	0.047*	
C12	0.1823 (6)	0.4436 (3)	0.1157 (5)	0.0304 (12)	
H12A	0.1626	0.4276	0.1832	0.037*	
C13	−0.0423 (5)	0.4099 (2)	0.3623 (4)	0.0148 (9)	
C14	−0.0838 (6)	0.3403 (3)	0.3060 (5)	0.0251 (11)	
C15	−0.061 (2)	0.3205 (7)	0.2026 (15)	0.067 (2)	0.47
H15	−0.0141	0.3488	0.1647	0.080*	0.47
C16	−0.110 (2)	0.2552 (6)	0.1541 (16)	0.067 (2)	0.47
H16	−0.1138	0.2452	0.0748	0.080*	0.47
N3	−0.1491 (7)	0.2101 (3)	0.2112 (6)	0.0647 (19)	
C17	−0.186 (2)	0.2304 (7)	0.3059 (16)	0.067 (2)	0.47
H17	−0.2300	0.1996	0.3416	0.080*	0.47
C18	−0.1591 (19)	0.2972 (7)	0.3543 (17)	0.067 (2)	0.47
H18	−0.1916	0.3112	0.4162	0.080*	0.47
C15'	0.0235 (16)	0.3043 (6)	0.2749 (17)	0.067 (2)	0.53
H15'	0.1159	0.3229	0.2870	0.080*	0.53
C16'	−0.0161 (15)	0.2386 (6)	0.2247 (17)	0.067 (2)	0.53
H16'	0.0504	0.2144	0.2003	0.080*	0.53
C17'	−0.2435 (15)	0.2459 (6)	0.2278 (17)	0.067 (2)	0.53
H17'	−0.3388	0.2285	0.2064	0.080*	0.53
C18'	−0.2165 (14)	0.3119 (6)	0.2774 (17)	0.067 (2)	0.53
H18'	−0.2923	0.3356	0.2901	0.080*	0.53

### Atomic displacement parameters (Å^2^)

**Table:**

	*U*^11^	*U*^22^	*U*^33^	*U*^12^	*U*^13^	*U*^23^
Tb1	0.01824 (16)	0.01848 (16)	0.01805 (16)	−0.00263 (9)	0.00676 (11)	−0.00260 (9)
O1	0.0188 (17)	0.0176 (17)	0.040 (2)	−0.0057 (14)	0.0135 (16)	−0.0055 (15)
O2	0.029 (2)	0.0208 (18)	0.037 (2)	0.0011 (16)	0.0197 (17)	−0.0011 (16)
O3	0.034 (2)	0.031 (2)	0.0143 (17)	−0.0008 (15)	0.0121 (16)	−0.0015 (14)
O4	0.0171 (19)	0.060 (3)	0.0183 (19)	−0.0084 (17)	0.0044 (16)	−0.0052 (17)
O5	0.0218 (18)	0.0230 (18)	0.0260 (19)	−0.0100 (15)	0.0092 (15)	−0.0068 (15)
O6	0.0246 (18)	0.0276 (19)	0.035 (2)	0.0016 (16)	0.0164 (17)	−0.0087 (17)
O7	0.0292 (19)	0.0203 (18)	0.0194 (17)	−0.0051 (15)	−0.0019 (15)	0.0041 (14)
O8	0.0188 (17)	0.035 (2)	0.029 (2)	0.0054 (15)	0.0126 (15)	0.0136 (16)
N2	0.029 (3)	0.053 (3)	0.017 (2)	0.008 (2)	−0.001 (2)	−0.008 (2)
C1	0.019 (2)	0.013 (2)	0.023 (3)	−0.0017 (19)	0.004 (2)	0.0002 (18)
C2	0.021 (2)	0.023 (2)	0.032 (3)	−0.008 (2)	0.006 (2)	−0.007 (2)
C3	0.074 (5)	0.041 (3)	0.094 (5)	−0.032 (3)	0.050 (4)	−0.034 (3)
C4	0.074 (5)	0.041 (3)	0.094 (5)	−0.032 (3)	0.050 (4)	−0.034 (3)
N1	0.069 (4)	0.037 (3)	0.097 (5)	−0.030 (3)	0.041 (4)	−0.040 (3)
C5	0.074 (5)	0.041 (3)	0.094 (5)	−0.032 (3)	0.050 (4)	−0.034 (3)
C6	0.074 (5)	0.041 (3)	0.094 (5)	−0.032 (3)	0.050 (4)	−0.034 (3)
C3'	0.074 (5)	0.041 (3)	0.094 (5)	−0.032 (3)	0.050 (4)	−0.034 (3)
C4'	0.074 (5)	0.041 (3)	0.094 (5)	−0.032 (3)	0.050 (4)	−0.034 (3)
C5'	0.074 (5)	0.041 (3)	0.094 (5)	−0.032 (3)	0.050 (4)	−0.034 (3)
C6'	0.074 (5)	0.041 (3)	0.094 (5)	−0.032 (3)	0.050 (4)	−0.034 (3)
C7	0.022 (3)	0.026 (3)	0.011 (2)	0.000 (2)	0.004 (2)	−0.0010 (19)
C8	0.018 (2)	0.031 (3)	0.010 (2)	0.004 (2)	0.005 (2)	−0.001 (2)
C9	0.027 (3)	0.047 (3)	0.014 (2)	−0.001 (2)	0.010 (2)	−0.001 (2)
C10	0.036 (3)	0.056 (4)	0.012 (3)	0.012 (3)	0.007 (2)	0.001 (2)
C11	0.023 (3)	0.055 (4)	0.035 (3)	−0.010 (3)	0.005 (3)	−0.011 (3)
C12	0.025 (3)	0.046 (3)	0.019 (3)	−0.005 (3)	0.008 (2)	−0.003 (2)
C13	0.017 (2)	0.012 (2)	0.011 (2)	−0.0018 (18)	0.0005 (18)	−0.0014 (17)
C14	0.021 (2)	0.020 (2)	0.031 (3)	−0.003 (2)	0.006 (2)	−0.009 (2)
C15	0.061 (4)	0.039 (3)	0.108 (6)	−0.021 (3)	0.041 (4)	−0.043 (4)
C16	0.061 (4)	0.039 (3)	0.108 (6)	−0.021 (3)	0.041 (4)	−0.043 (4)
N3	0.063 (4)	0.032 (3)	0.084 (5)	−0.016 (3)	0.011 (4)	−0.031 (3)
C17	0.061 (4)	0.039 (3)	0.108 (6)	−0.021 (3)	0.041 (4)	−0.043 (4)
C18	0.061 (4)	0.039 (3)	0.108 (6)	−0.021 (3)	0.041 (4)	−0.043 (4)
C15'	0.061 (4)	0.039 (3)	0.108 (6)	−0.021 (3)	0.041 (4)	−0.043 (4)
C16'	0.061 (4)	0.039 (3)	0.108 (6)	−0.021 (3)	0.041 (4)	−0.043 (4)
C17'	0.061 (4)	0.039 (3)	0.108 (6)	−0.021 (3)	0.041 (4)	−0.043 (4)
C18'	0.061 (4)	0.039 (3)	0.108 (6)	−0.021 (3)	0.041 (4)	−0.043 (4)

### Geometric parameters (Å, °)

**Table:**

Tb1—O3	2.426 (4)	C6—H6	0.9300
Tb1—O6^i^	2.432 (3)	C3'—C4'	1.393 (14)
Tb1—O5	2.446 (3)	C3'—H3'	0.9300
Tb1—O2^ii^	2.466 (3)	C4'—H4'	0.9300
Tb1—O1	2.483 (3)	C5'—C6'	1.410 (14)
Tb1—O4^ii^	2.491 (4)	C5'—H5'	0.9300
Tb1—O7	2.530 (3)	C6'—H6'	0.9300
Tb1—O8	2.561 (3)	C7—C8	1.495 (7)
O1—C1	1.250 (6)	C8—C12	1.365 (7)
O2—C1	1.249 (5)	C8—C9	1.414 (7)
O2—Tb1^ii^	2.466 (3)	C9—C10	1.395 (8)
O3—C7	1.258 (6)	C9—H9A	0.9300
O4—C7	1.250 (7)	C10—H10A	0.9300
O4—Tb1^ii^	2.491 (4)	C11—C12	1.383 (7)
O5—C13	1.247 (6)	C11—H11A	0.9300
O6—C13	1.232 (6)	C12—H12A	0.9300
O6—Tb1^i^	2.432 (3)	C13—C14	1.513 (6)
O7—H71	0.8413	C14—C18'	1.329 (12)
O7—H72	0.8403	C14—C15	1.360 (13)
O8—H81	0.8508	C14—C18	1.374 (14)
O8—H82	0.8460	C14—C15'	1.415 (12)
N2—C10	1.314 (8)	C15—C16	1.421 (14)
N2—C11	1.316 (8)	C15—H15	0.9300
C1—C2	1.513 (7)	C16—N3	1.252 (14)
C2—C3'	1.356 (13)	C16—H16	0.9300
C2—C6	1.357 (12)	N3—C17'	1.230 (12)
C2—C6'	1.367 (13)	N3—C17	1.338 (14)
C2—C3	1.368 (12)	N3—C16'	1.362 (13)
C3—C4	1.411 (13)	C17—C18	1.423 (14)
C3—H3	0.9300	C17—H17	0.9300
C4—N1	1.261 (12)	C18—H18	0.9300
C4—H4	0.9300	C15'—C16'	1.419 (13)
N1—C4'	1.259 (13)	C15'—H15'	0.9300
N1—C5	1.331 (13)	C16'—H16'	0.9300
N1—C5'	1.349 (13)	C17'—C18'	1.412 (13)
C5—C6	1.401 (13)	C17'—H17'	0.9300
C5—H5	0.9300	C18'—H18'	0.9300
			
O3—Tb1—O6^i^	147.12 (12)	C2—C3'—H3'	120.1
O3—Tb1—O5	84.03 (12)	C4'—C3'—H3'	120.1
O6^i^—Tb1—O5	95.50 (12)	N1—C4'—C3'	124.5 (13)
O3—Tb1—O2^ii^	70.56 (12)	N1—C4'—H4'	117.8
O6^i^—Tb1—O2^ii^	142.13 (12)	C3'—C4'—H4'	117.8
O5—Tb1—O2^ii^	82.30 (12)	N1—C5'—C6'	123.8 (12)
O3—Tb1—O1	80.33 (11)	N1—C5'—H5'	118.1
O6^i^—Tb1—O1	78.94 (12)	C6'—C5'—H5'	118.1
O5—Tb1—O1	139.44 (12)	C2—C6'—C5'	116.9 (12)
O2^ii^—Tb1—O1	125.85 (13)	C2—C6'—H6'	121.6
O3—Tb1—O4^ii^	120.33 (13)	C5'—C6'—H6'	121.6
O6^i^—Tb1—O4^ii^	79.95 (13)	O4—C7—O3	123.8 (5)
O5—Tb1—O4^ii^	140.54 (12)	O4—C7—C8	117.5 (4)
O2^ii^—Tb1—O4^ii^	78.39 (12)	O3—C7—C8	118.7 (5)
O1—Tb1—O4^ii^	78.61 (12)	C12—C8—C9	118.1 (5)
O3—Tb1—O7	77.28 (12)	C12—C8—C7	122.1 (5)
O6^i^—Tb1—O7	71.90 (12)	C9—C8—C7	119.8 (5)
O5—Tb1—O7	69.43 (12)	C10—C9—C8	117.2 (6)
O2^ii^—Tb1—O7	138.97 (12)	C10—C9—H9A	121.4
O1—Tb1—O7	70.70 (11)	C8—C9—H9A	121.4
O4^ii^—Tb1—O7	141.47 (12)	N2—C10—C9	124.4 (5)
O3—Tb1—O8	136.28 (12)	N2—C10—H10A	117.8
O6^i^—Tb1—O8	72.55 (12)	C9—C10—H10A	117.8
O5—Tb1—O8	70.81 (12)	N2—C11—C12	124.2 (5)
O2^ii^—Tb1—O8	71.07 (11)	N2—C11—H11A	117.9
O1—Tb1—O8	140.79 (12)	C12—C11—H11A	117.9
O4^ii^—Tb1—O8	70.46 (12)	C8—C12—C11	118.9 (5)
O7—Tb1—O8	122.57 (11)	C8—C12—H12A	120.5
C1—O1—Tb1	135.8 (3)	C11—C12—H12A	120.5
C1—O2—Tb1^ii^	135.9 (3)	O6—C13—O5	125.9 (4)
C7—O3—Tb1	171.2 (4)	O6—C13—C14	117.5 (4)
C7—O4—Tb1^ii^	107.9 (3)	O5—C13—C14	116.7 (4)
C13—O5—Tb1	134.8 (3)	C18'—C14—C15	96.9 (10)
C13—O6—Tb1^i^	173.6 (3)	C18'—C14—C18	39.1 (10)
Tb1—O7—H71	122.2	C15—C14—C18	118.0 (9)
Tb1—O7—H72	126.1	C18'—C14—C15'	117.3 (8)
H71—O7—H72	102.6	C15—C14—C15'	41.4 (9)
Tb1—O8—H81	122.0	C18—C14—C15'	110.2 (9)
Tb1—O8—H82	120.6	C18'—C14—C13	124.7 (7)
H81—O8—H82	106.6	C15—C14—C13	122.8 (7)
C10—N2—C11	117.1 (5)	C18—C14—C13	119.0 (7)
O2—C1—O1	127.3 (5)	C15'—C14—C13	117.9 (6)
O2—C1—C2	115.6 (4)	C14—C15—C16	118.1 (13)
O1—C1—C2	117.1 (4)	C14—C15—H15	120.9
C3'—C2—C6	98.8 (10)	C16—C15—H15	120.9
C3'—C2—C6'	118.3 (8)	N3—C16—C15	124.2 (14)
C6—C2—C6'	42.0 (9)	N3—C16—H16	117.9
C3'—C2—C3	42.0 (9)	C15—C16—H16	117.9
C6—C2—C3	117.2 (8)	C17'—N3—C16	93.6 (12)
C6'—C2—C3	105.6 (10)	C17'—N3—C17	41.9 (11)
C3'—C2—C1	123.0 (7)	C16—N3—C17	116.6 (9)
C6—C2—C1	122.2 (7)	C17'—N3—C16'	118.1 (9)
C6'—C2—C1	118.7 (7)	C16—N3—C16'	46.0 (10)
C3—C2—C1	120.5 (6)	C17—N3—C16'	108.1 (10)
C2—C3—C4	118.3 (11)	N3—C17—C18	122.7 (13)
C2—C3—H3	120.8	N3—C17—H17	118.6
C4—C3—H3	120.8	C18—C17—H17	118.6
N1—C4—C3	124.6 (12)	C14—C18—C17	117.5 (12)
N1—C4—H4	117.7	C14—C18—H18	121.3
C3—C4—H4	117.7	C17—C18—H18	121.3
C4'—N1—C4	43.1 (10)	C14—C15'—C16'	117.1 (11)
C4'—N1—C5	95.7 (11)	C14—C15'—H15'	121.4
C4—N1—C5	117.2 (8)	C16'—C15'—H15'	121.4
C4'—N1—C5'	116.3 (9)	N3—C16'—C15'	121.7 (11)
C4—N1—C5'	104.1 (10)	N3—C16'—H16'	119.1
C5—N1—C5'	44.9 (10)	C15'—C16'—H16'	119.1
N1—C5—C6	121.8 (12)	N3—C17'—C18'	124.3 (12)
N1—C5—H5	119.1	N3—C17'—H17'	117.9
C6—C5—H5	119.1	C18'—C17'—H17'	117.9
C2—C6—C5	119.5 (11)	C14—C18'—C17'	120.6 (11)
C2—C6—H6	120.2	C14—C18'—H18'	119.7
C5—C6—H6	120.2	C17'—C18'—H18'	119.7
C2—C3'—C4'	119.7 (12)		

Symmetry codes: (i) −*x*, −*y*+1, −*z*+1; (ii) −*x*+1, −*y*+1, −*z*+1.

### Hydrogen-bond geometry (Å, °)

**Table:**

*D*—H···*A*	*D*—H	H···*A*	*D*···*A*	*D*—H···*A*
O7—H71···N3^iii^	0.84	1.96	2.787 (7)	168.
O7—H72···N2^iv^	0.84	1.98	2.802 (5)	167.
O8—H81···N1^v^	0.85	2.01	2.837 (7)	163.
O8—H82···O7^i^	0.85	2.16	3.002 (5)	171.

Symmetry codes: (iii) −*x*, *y*+1/2, −*z*+1/2; (iv) −*x*, −*y*+1, −*z*; (v) −*x*+1, *y*−1/2, −*z*+3/2; (i) −*x*, −*y*+1, −*z*+1.
